# The Circulating Treg/Th17 Cell Ratio Is Correlated with Relapse and Treatment Response in Pulmonary Sarcoidosis Patients after Corticosteroid Withdrawal

**DOI:** 10.1371/journal.pone.0148207

**Published:** 2016-02-04

**Authors:** Yongzhe Liu, Lan Qiu, Yanxun Wang, Halimulati Aimurola, Yuyue Zhao, Shan Li, Zuojun Xu

**Affiliations:** 1 Department of Respiratory Medicine, Peking Union Medical College Hospital, Chinese Academy of Medical Science & Peking Union Medical College, Beijing 100730, China; 2 Department of Internal Medicine, No. 2 Jikun Hospital, Urumchi, Xinjiang Uyghur Autonomous Region 830013, China; 3 Department of Respiratory Medicine, Chest Hospital of Xinjiang Uyghur Autonomous Region, Urumchi, Xinjiang Uyghur Autonomous Region 830049, China; Universitatsklinikum Freiburg, GERMANY

## Abstract

**Objectives:**

Pulmonary sarcoidosis is an immune-mediated disease, and some patients can be effectively treated with corticosteroids. However, nearly half of all sarcoidosis patients relapse after corticosteroid withdrawal. Different subsets of CD4+ helper T cells participate in the immunopathogenesis of sarcoidosis. Thus, the aims of our study were to investigate whether the circulating subsets of CD4+ helper T cells were associated with sarcoidosis relapse and with its remission after retreatment. Additionally, we identified a useful biomarker for predicting the relapse and remission of sarcoidosis patients.

**Methods:**

Forty-two patients were enrolled in the present study who had previously been diagnosed with pulmonary sarcoidosis and treated with corticosteroids. The patients were allocated into either a stable group if they exhibited sustained remission (n = 22) or a relapse group if they experienced clinical or radiological recurrence after treatment withdrawal (n = 20). Peripheral blood cells were collected from these patients and analyzed to determine the frequencies of subsets of circulating CD4+ helper T cells by flow cytometry. The patients in the relapse group were retreated with corticosteroids and immunosuppressive agents and were then reevaluated to determine the frequencies of dynamic subsets of circulating CD4+ helper T cells after remission.

**Results:**

The frequencies of circulating Tregs were significantly increased concomitant with a decrease in the circulating Th17 cell frequency in the relapsed patients compared with the stable patients. The Treg/Th17 ratio was negatively correlated with sarcoidosis activity and was sensitive to retreatment. In addition, the percentage of isolated CD45RO+Ki67+ Tregs was higher in the patients who were stable and in those who recovered after retreatment than in those who relapsed.

**Conclusions:**

An imbalance between Tregs and Th17 cells is associated with pulmonary sarcoidosis relapse after corticosteroid withdrawal. The circulating Treg/Th17 ratio could serve as an alternative marker for monitoring pulmonary sarcoidosis relapse after the end of corticosteroid treatment and for rapidly predicting the response to retreatment.

## Introduction

Sarcoidosis is multisystemic disease of unknown etiology. It usually involves the respiratory tract and is characterized by the formation of granulomas. This disease has shown an increased prevalence and incidence in recent years, indicating that it might be more common than previously believed [1,2]. It has a benign course, and more than half of all cases spontaneously recover. However, some active multisystemic and persistent sarcoidosis cases may evolve into chronic disease without pharmaceutical therapy, leading to pulmonary fibrosis and a decline of pulmonary function over the long term, resulting in morbidity and mortality [3].

To our knowledge, sarcoidosis is a sustained immune-mediated disease that causes inflammatory activity in local organs and the progression of granulomatous formation. Although pulmonary sarcoidosis might never cause pulmonary symptoms and can resolve within months, it is often a chronic disease lasting for more than one year. The exact reason why sarcoidosis spontaneously resolves in some patients and progresses in others is poorly understood. Multiple factors account for the outcomes and dissemination of sarcoidosis. Differences in genetic backgrounds, immunological responses and causative agents that are as yet unrecognized could affect the outcomes of syndromes, and these factors require further elucidation and new therapeutic approaches [4]. Upon activation and expansion, CD4+ T cells develop into different T helper subsets with different cytokine profiles and distinct effector functions [5]. Previous reports have delineated how CD4+ helper T cell subsets, such as Th1, Th2, Th17 and regulatory T cells, cooperate or interfere with each other to orchestrate the progression or control of sarcoidosis [6].

An uncontrolled Th1 immune response occurring in organs affected by the disease has been shown to be a key mechanism in the initiation and maintenance of granulomatous inflammation. Th1 cells mainly produce the cytokine interferon-γ, which is predominant in sarcoidosis [7]. Th2 cells are defined as the subset that produces the cytokine IL-4, which limits inflammation in the process of granulomatous formation [8]. A switch from the typical Th1 immune response toward the production of Th2 cytokines has been suggested to be important for the further development of fibrosis [9]. Sarcoidosis is also associated with dysfunction of both peripheral and intratissue regulatory T cells. Recent studies have focused on the roles of Treg cells in maintaining immune homeostasis and preventing autoimmunity, demonstrating that Tregs suppress the initial steps of granulomatous formation in sarcoidosis [10,11]. However, the number of Tregs involved in the process of granuloma formation is unknown. Additionally, Th17 cells produce the potent proinflammatory cytokine IL-17A, which plays a crucial role in sarcoidosis-associated inflammation [12]. The finding of increased numbers of Th17 cells in the bronchoalveolar lavage fluid (BALF), blood and granulomatous tissue of sarcoidosis patients suggests that these cells contribute to the pathogenesis of sarcoidosis [13]. Further evidence has shown that Tregs and Th17 cells mutually influence the pathogenesis of sarcoidosis. An imbalance in Tregs and Th17 cells has a role in non-specific tissue inflammation and has been associated with the pathogenesis of several autoimmune diseases and viral infectious conditions [14–16]. In our previous work, we have demonstrated imbalances in Treg cells and Th17 cells in the peripheral blood and BALF of sarcoidosis patients compared with healthy controls [17]. Treg frequencies in the blood and BALF tended to decrease in patients with active sarcoidosis, accompanied by an increase in Th17 cells.

The ideal goals of sarcoidosis treatment are the control of symptoms, improvement of impaired pulmonary function and reduction in the dosage of corticosteroids [18]. In China, the first-line option for treating pulmonary sarcoidosis is oral steroids, and the second-line option is immunosuppressive agents [19]. Most patients achieve clinical and radiographic improvement after a round of medications, but prolonged use of steroids or immunosuppressive agents has been associated with various adverse effects, and intolerance following long-term treatment decreases compliance and quality of life [20]. In contrast, relapse occurs occasionally when medications are tapered or withdrawn, with sarcoidosis relapse rates ranging from 13% to 75% in different populations [21]. These relapses typically occur one month to one year after therapy is tapered or discontinued. None of the alternative biomarkers that have been identified to date have been demonstrated to predict relapse after the end of treatment, and there are no optimized predictions based on changes in immunological signatures that can be used to guide treatment. Available markers and tests for active granulomatous inflammation in sarcoidosis, including serum angiotensin-converting enzyme (sACE) [22], gallium-67 scanning and bronchoalveolar lavage analysis, are unfortunately not suitable for predicting relapse. In particular, gallium-67 uptake is rapidly suppressed by glucocorticoids, independent of the effects of glucocorticoids on sarcoidosis itself [23]. Management of these patients by clinicians is complicated because there are no biomarkers for predicting relapse after the end of treatment and during the follow-up period. Most studies have focused on differences in the immune status between sarcoidosis patients and healthy controls, and only limited data are available on dynamic immunity in relapsed and stable sarcoidosis patients [24,25]. Therefore, we were interested in identifying the different CD4+ helper T cells that might mediate the pathogenesis of relapsing and stable pulmonary sarcoidosis to detect a promising biomarker related to the relapse of sarcoidosis.

In the present study, we performed intercellular cytoplasmic cytokine staining (ICCS) of CD4+ helper T cells and aimed to examine the different subsets of these cells during the courses of relapsing and remitting pulmonary sarcoidosis in patients after corticosteroid withdrawal. This study was also designed to investigate the dynamics of the Treg/Th17 ratio in pulmonary sarcoidosis patients after corticosteroid withdrawal, and we attempted to establish the utility of the Treg/Th17 ratio for prediction of the relapse of sarcoidosis patients and the outcomes of retreatment.

## Methods

### Patients and sample collection

Patients who were consecutively enrolled as respiratory outpatients at the Interstitial Lung Disease Center of Peking Union Medical College Hospital and who were formerly diagnosed with pulmonary sarcoidosis from December 2013 to December 2014 were included. This center provides national diagnosis and therapy for interstitial lung diseases, particularly sarcoidosis. The study was approved by the Ethics Committee of Peking Union Medical Hospital. All of the patients were informed about the details of this study, and they signed consent forms before enrollment. The criteria for pulmonary sarcoidosis in this study were as follows: typical high-resolution computed tomography (HRCT) images showing pulmonary nodules and/or bilateral mediastinal lymphadenopathy; clinical manifestations, such as fatigue, coughing, and weight loss; and histological confirmation of noncaseating granulomas by transbronchial needle aspiration (TBNA) or transbronchial lung biopsy (TBLB). The following patients were excluded from the study: those with relapsed pulmonary sarcoidosis who were refractory to corticosteroids and methotrexate (MTX) therapy; those with progressive sarcoidosis with pulmonary fibrosis and acute respiratory conditions, such as confirmed infection with exogenous pathogens; and those who took ACE inhibitors.

For the purposes of this study, the pulmonary sarcoidosis patients were divided into the following two groups (S1 Fig): a stable group, in which the patients exhibited with sustained remission after corticosteroid withdrawal, and a relapse group, consisting of patients with clinical manifestations or radiographic recurrence after corticosteroid withdrawal who needed retreatment. The retreatment schedule consisted of prednisone and MTX. The initial dosage of prednisone was 1 mg/kg (maximum of 40 mg), and the dosage was tapered biweekly to 10 mg for maintenance. MTX was started at 10 mg weekly and was assessed based on whole blood cell counts and hepatic function every 2 weeks.

The medical histories of the patients were collected, and symptoms were recorded. Additionally, at enrollment and at the 12-week time point, blood samples were collected for laboratory tests, such as sACE, whole blood analysis, flow cytometry (FCM) analysis and pulmonary function tests (PFTs). Sarcoidosis stage was defined according to the Scadding staging system at enrollment. The PFT parameters recorded included the percentage of predicted forced expiratory volume in one second (FEV1%) and the diffusing capacity of the lungs for carbon monoxide (DLco%). All of the relapse patients were reevaluated to determine the stage of sarcoidosis and the changes in HRCT findings after retreatment.

### Antibodies and reagents

Fluorescein isothiocyanate (FITC)-conjugated anti-Foxp3 and phycoerythrin (PE)-conjugated anti-IL-17A monoclonal antibodies (mAbs) were purchased from eBioscience (San Diego, CA, USA). Peridinin chlorophyll protein (PerCP)-anti-CD8, anti-CD4, FITC-anti-IFN-γ, PE-anti-IL-4, anti-CD25, anti-Ki67 and allophycocyanin (APC)-anti-CD3, anti-CD45RO mAbs were purchased from BD Biosciences (San Jose, CA, USA). Phorbol-12-myristate-13-acetate (PMA), brefeldin and ionomycin were acquired from Sigma-Aldrich (St. Louis, MO, USA).

### Intracellular ICCS

Freshly heparinized peripheral blood samples collected from sarcoidosis patients were aliquoted into tubes, and 200-μl samples were incubated with mAbs for 20 minutes in darkness for surface staining; duplicate samples with the corresponding antibody isotype controls served as negative controls. The samples were then processed with red blood cell lysing solution (BD Pharmingen, CA, USA) and were washed twice with phosphate-buffered saline (PBS, Gibco, USA). For ICCS analysis, each sample was fixed and permeabilized (Fix/Perm Kit, eBioscience, USA) according to the manufacturer’s instructions; then, the cells were incubated with anti-Foxp3 mAb for 20 minutes in darkness, washed twice with PBS, and fixed with 1% paraformaldehyde. For Th1, Th2, and Th17 analyses, each 200-μl aliquot of freshly heparinized peripheral blood was cultured with 800 μl of RPMI-1640 supplemented with 10% heat-inactivated fetal calf serum (FCS) for 5 hours, in addition to 300 ng/ml PMA and 1 μl/ml ionomycin. At the first hour, 1 μl of GolgiStop^™^ (BD Pharmingen, CA, USA) was added to each sample. Subsequently, each sample was processed following the ICCS procedure mentioned above and was finally incubated with anti-IFN-γ, anti-IL-4 and anti-IL-17A mAbs separately, according to the manufacturers' instructions. Flow cytometric analysis was performed using a FACS Calibur flow cytometer (Becton Dickinson, USA), and the percentages of each cell type were analyzed using Flow Jo software (Tree Star, OR, USA).

### Isolation of CD4+CD25+ regulatory T cells

Freshly heparinized blood samples from sarcoidosis patients were two-fold diluted with RPMI-1640 containing 10% FCS. Peripheral blood mononuclear cells (PBMCs) were isolated by gradient centrifugation using Ficoll-Paque^™^ Plus (GE Healthcare Biosciences, Sweden) and were resuspended in RPMI-1640 medium supplemented with 300 μg/ml L-glutamine, 100 U/ml penicillin, 100 μg/ml streptomycin, and 10% FCS. Isolation of CD4+CD25+ T cells was performed using a human regulatory T cell isolation kit (Miltenyi Biotec, CA, USA), and the cells were separated by magnetic-activated cell sorting (MACS) according to the manufacturer’s instructions. The isolated Tregs were then cultured with recombinant human IL-2 (50 IU/ml, Peprotech, UK) in 24-well plates (Corning, USA) at a density of 5×10^5^ cells per ml. Anti-Ki-67 mAb was applied following the ICCS procedure described above, and the cells were analyzed by FCM.

### Statistical analysis

GraphPad Prism software version 5.0 (San Diego, CA, USA) was used for all statistical analyses. The data are presented as the mean ± standard deviation (SD). The Mann-Whitney U-test was applied to comparisons between two groups. Correlations between two variables were evaluated using Spearman’s rank test. Data from the same individuals were compared using Wilcoxon's matched-pairs test. For all of the tests, a *P* value of less than 0.05 was considered to indicate a significant difference.

## Results

### 1. Characteristics of the patients enrolled in this study

To evaluate the differences between relapsed and stable patients, we enrolled a total of 42 pulmonary sarcoidosis patients in this study with a history of corticosteroid treatment, all of whom were classified as stage 2 according to the Scadding score system [26]. Twenty-two patients were allocated to the stable group after prednisone withdrawal, and twenty were allocated to the relapse group after the end of corticosteroid withdrawal. According to the clinical data listed in Table 1, the majority of the enrolled sarcoidosis patients were female (83%), 11 (26%) had a history of smoking, the duration of corticosteroid usage in the stable group (18.3±10.3 months) was longer than that in the relapse group (14.5±8.7 months, p<0.01), and more relapse patients had extrapulmonary involvement of organs such as the skin or eyes. Inflammatory activity indices, such as highly sensitive C-reactive protein (CRP; 7.9±5.7 vs. 6.15±4.7, p = 0.39) and ESR (40.9±16.8 vs. 30.77±12.6, p = 0.043), were higher in the relapse group. The sACE level in the stable group (32.7±9.1 U/L) was lower than that in the relapse group (54.7±14.8 U/L, p<0.01). Regarding the PFT results, FEV1 (83.9±8.2% vs. 92.4±6.42%, p<0.01) and DLco (80.79±7.48% vs. 88.6±4.81%, p<0.01) (presented as percentages of the predicted values) were lower in the relapse group compared with the stable group.

**Table 1:** Clinical characteristics of the enrolled sarcoidosis patients (n = 42).

	Stable group	Relapse group	P-value
No. of patients	22	20	
Age	44.1±12.2	43.2±8.5	0.51
Sex (male/female)	4/18	3/17	
Duration of previous prednisone (months)	18.3±10.3	14.5±8.7	<0.01
Smoking history*	5	6	
Extrapulmonary involvement			
Cardiac/skin/ocular	1/2/2	2/5/3	
Laboratory tests			
ESR (mm/h)	30.77±12.6	40.9±16.8	0.043
hsCRP (mg/l)	6.15±4.7	7.9±5.7	0.39
Serum ACE (U/L)	32.7±9.1	54.7±14.8	<0.01
Pulmonary function			
FEV1% pred	92.4±6.42	83.9±8.2	<0.01
DLCO% pred	88.6±4.81	80.79±7.4	<0.01

* All of the patients who smoked were forbidden to smoke during the follow-up period

Data are presented as the mean ± standard deviation

### 2. The frequencies of Th1, Th2, and Th17 CD4+ helper T cells, Treg cells, and the Treg/Th17 ratio in the peripheral blood of relapsed and stable sarcoidosis patients after corticosteroid withdrawal

**Fig 1:**
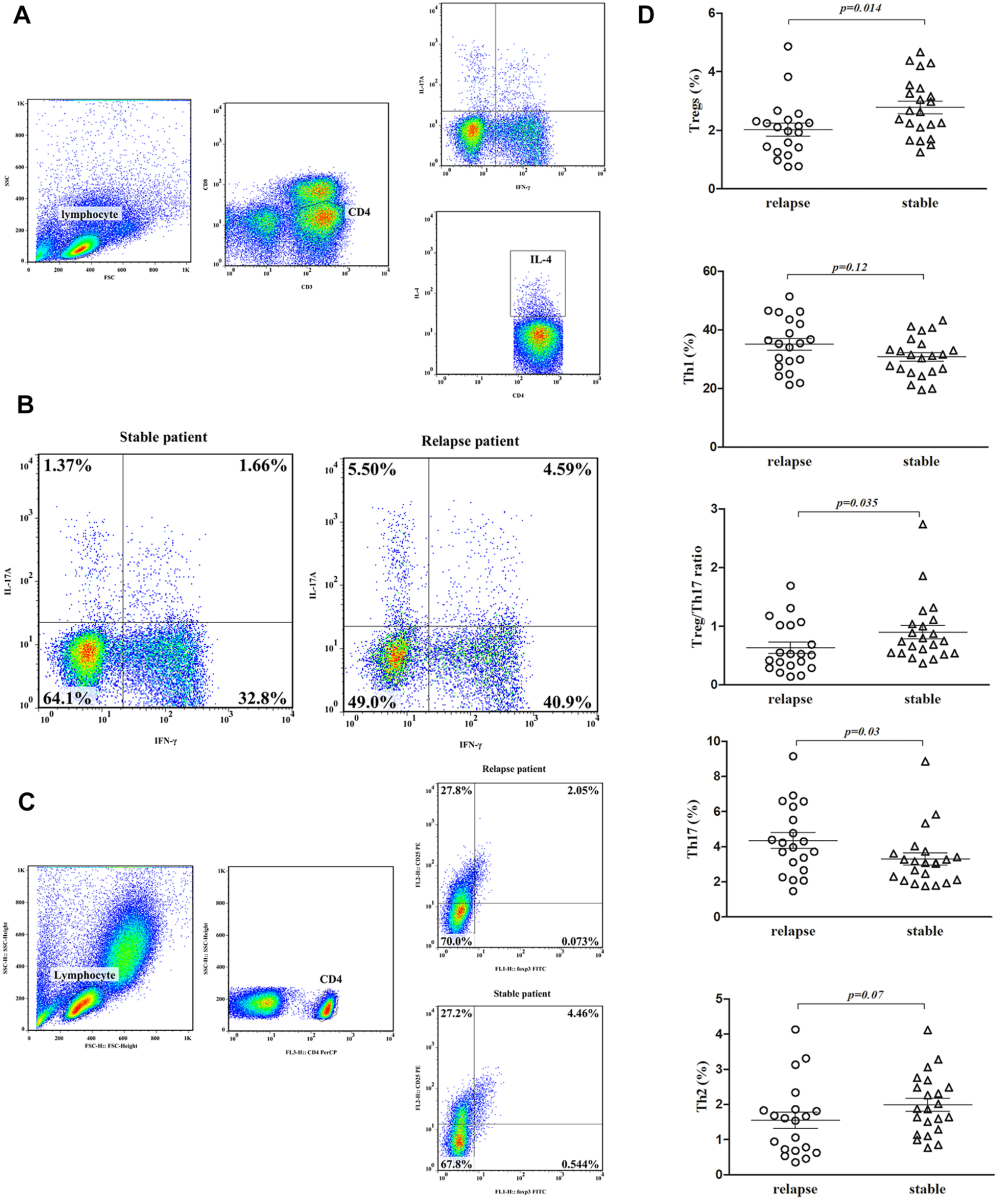
The distributions of Th1, Th2, Th17 and Treg cells in the peripheral blood in relapsed and stable pulmonary sarcoidosis patients after corticosteroid withdrawal. IFN-γ+CD4+ T cells were defined as Th1 cells, IL-4+CD4+ T cells were defined as Th2 cells, IL-17A+CD4+ T cells were defined as Th17 cells, and Foxp3+CD25+CD4+ T cells were defined as Treg cells. (A) Gating strategy for analysis of IL-17A-, IFN-γ-, and IL-4-expressing CD4+ T cells sorted from lymphocytes by ICCS. The values in the quadrants indicate the percentages of each CD4+ T cell subset. (B) Representative dot plots of Th17 and Th1 cells in the peripheral blood of relapsed and stable patients. (C) Gating strategy for analysis of Foxp3+CD25+ cells in the CD4+ T population sorted from lymphocytes by ICCS. CD25+Foxp3+ cells were gated to correspond to the isotype control. (D) Pooled data demonstrating the percentages of gated Th1, Th2, Th17 and Treg cells from CD4+ T cells in relapsed and stable pulmonary sarcoidosis patients after corticosteroid withdrawal. The percentage of Treg cells and the Treg/Th17 ratio in the stable patients were higher than those in the relapse patients, while the percentage of Th17 cells was lower in the stable patients (p<0.05). There were no differences in the percentage of Th1 cells or Th2 cells between the two groups.

To assess the differences in CD4+ helper T cells between the relapsed and stable sarcoidosis patients, different CD4+ T cell subsets were gated according to the strategy shown in Fig 1A. The percentage of Th1 CD4+ T cells showed a slight increase in the relapse group (35.1±8.9%) compared with the stable group (30.8±6.8%), but this difference was not significant (p = 0.12, Fig 1B). The percentage of Th2 CD4+ T cells did not differ between the relapse group (1.54±1.0%) and the stable group (1.99±0.85%, p = 0.07). Compared with the stable patients, the relapsed patients showed an increased percentage of Th17 cells (4.35±1.9% vs. 3.3±1.6%, p = 0.03) accompanied by a decreased percentage of total Treg cells (2.03±0.9% vs. 2.7±1.0%, p = 0.014, Fig 1C). The Treg/Th17 ratio in the stable group was higher than that in the relapse group (0.89±0.54 vs. 0.63±0.43, p = 0.03). The gating results are shown in Fig 1D. Foxp3+CD25+CD4+ T cells showed the same trend as the total number of Tregs between the groups (2.31±1.0% vs. 1.56±0.8%, p = 0.013).

### 3. Evaluation of remission after retreatment in relapsed patients

Remission in the relapsed patients was evaluated by examining symptoms and HRCT, laboratory test and PFT results. The data from 16 patients who completed the three-month follow-up in the relapse group were analyzed, excluding data from two patients did not respond to retreatment and two patients who could not tolerate the side effects of the combined medications. As shown in Table 2, all relapsed patients recovered from intermittent cough, which was the main symptom of relapse, with or without remission of other pulmonary symptoms. Most relapsed patients with comorbid symptoms such as a mild fever, fatigue and organ manifestations recovered within three months after retreatment. In the relapsed patients, HRCT scans performed after three months of retreatment showed that lesions either in hilar or lung fields were remarkably improved, as determined by a respiratory doctor and a radiologist. Symmetrical scattered nodules were the dominant lesions in the relapsed patients, and they were mitigated after treatment with prednisone and MTX for three months. Laboratory tests indicated that inflammatory markers, such as hsCRP and sACE, were within the normal range in half of the relapsed patients, and in the patients with elevated levels of these markers, the levels returned to normal after retreatment. The relapsed sarcoidosis patients with obvious PFT impairment showed improvements in the predicted values of FEV1% and DLco% after retreatment.

**Table 2:** Clinical characteristics of remission patients who underwent retreatment (n = 16).

	Relapse	Remission
**Pulmonary Symptoms**		
Cough	16	0
Wheeze	12	2
Shortness of breath	9	1
**Comorbid Symptoms**		
Fever	4	0
Fatigue	10	1
Organ involvement	5	0
**HRCT Lesions**		
Symmetrical/Unilateral	11/5	2/1
Hilar/Field	16/12	8/4
Nodular/Pathy/Opaque	14/5/5	4/1/1
Scattered/Multiple	10/6	2/2
**Laboratory Tests**		
ESR elevated	13	3
hsCRP elevated	7	0
Serum ACE elevated	5	0
**Pulmonary Function Tests**		
Reduced FEV1%	6	0
Reduced DLco%	8	0

ESR elevated: above 15 mm/h in males and above 20 mm/h in females; hsCRP elevated: above 3.0 mg/l; serum ACE elevated: above 62 U/ml. Reduced FEV1 and DLco% were below 80 percent of the predicted values.

### 4. The dynamic frequencies of Th1, Th2, and Th17 CD4+ helper T cells, Treg cells and the Treg/Th17 ratio after 12 weeks of retreatment

**Fig 2:**
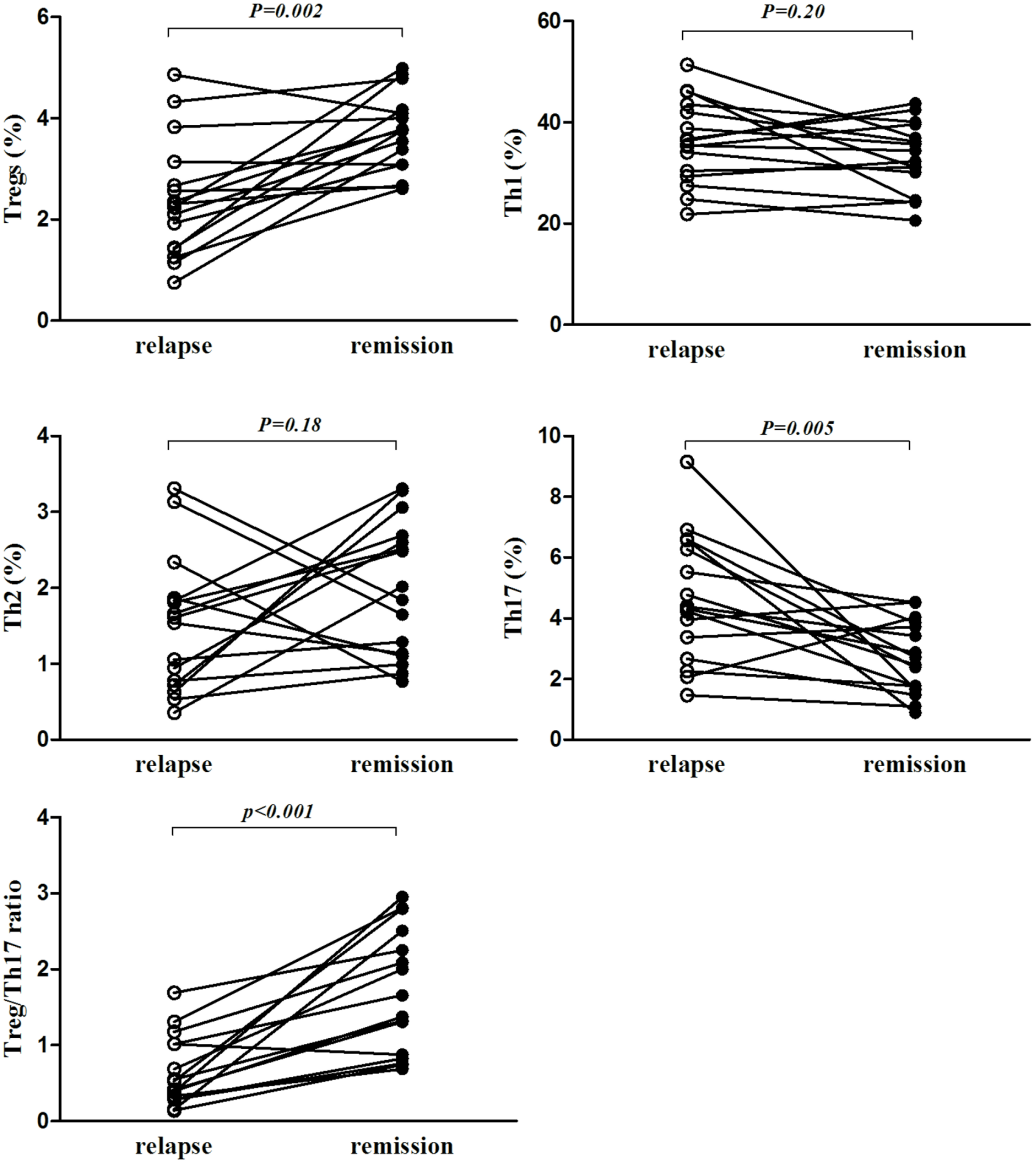
The percentages of Th1, Th2, Th17, and Treg cells at the onset of relapse and at remission after 12 weeks of retreatment. The percentage of Treg cells and the Treg/Th17 ratio were increased after retreatment in the relapsed sarcoidosis patients; this increase was accompanied by a decrease in the percentage of Th17 T cells (p<0.05). The percentages of Th1 and Th2 cells did not differ between the relapsed and remission patients.

To evaluate changes in the frequencies of Th1, Th2, and Th17 CD4+ helper cells in the relapsed patients, the patients in the relapse group were retreated with corticosteroids and immunosuppressive agents for 12 weeks, and they all achieved symptom remission or radiographic improvement. The proportions of different CD4+ helper T cell subsets after remission were analyzed by FCM. The percentage of the Th1 subset showed a decreasing trend after 12 weeks of retreatment (36.2±8.24% vs. 32.9±6.96%), but this difference was not significant (p = 0.2). The percentage of the Th2 subset did not differ between the relapse and remission groups (1.5±0.8% vs. 1.97±0.8%, p = 0.18). The percentage of the Th17 subset was decreased in the relapsed patients after three months of retreatment (4.65±2.0% vs. 2.7±1.2%, p = 0.005), accompanied by an increase in the Treg percentage during remission (2.39±1.16% vs. 3.7±0.77%, p = 0.002). The Treg/Th17 ratio in the relapse group increased dramatically after pulmonary sarcoidosis remission (0.65±0.45% vs. 1.68±0.8%, p<0.005, Fig 2).

### 5. The Treg/Th17 ratio is correlated with the severity of pulmonary sarcoidosis in relapsed patients

**Fig 3:**
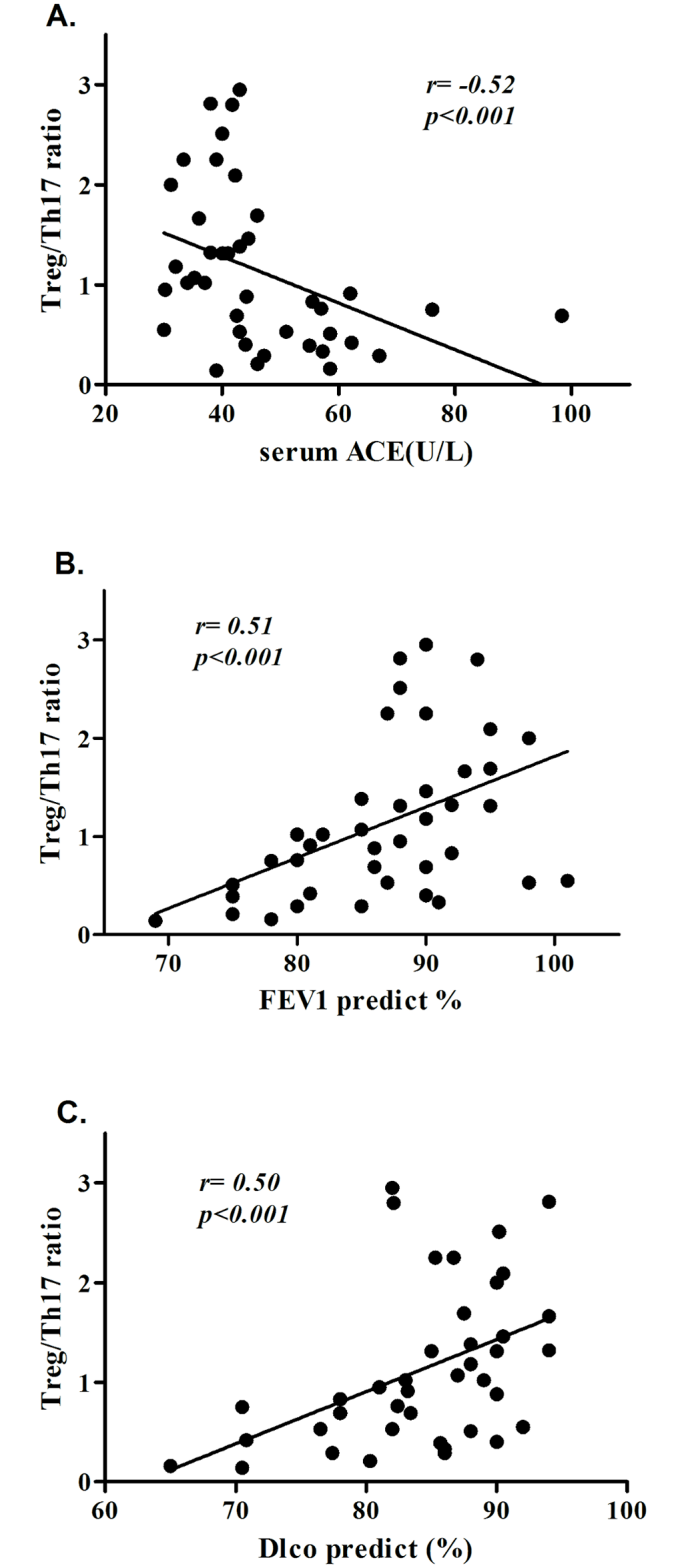
The Treg/Th17 ratio was correlated with serum ACE and FEV1% and DLco% predicted values in relapsed sarcoidosis patients. Each dot represents a value measured before or after retreatment. (A) The Treg/Th17 ratio and serum ACE showed a negative correlation in the relapsed patients.(B) The Treg/Th17 ratio demonstrated a moderate positive correlation with the FEV1% predicted value in the relapsed patients.(C) The Treg/Th17 ratio demonstrated a moderate positive correlation with the DLco% predicted value in the relapsed patients.

To better understand the associations between the Treg/Th17 ratio and clinical indices in sarcoidosis relapse patients, we assessed several useful indices, including serum ACE and FEV1% and DLco% predicted values. The Treg/Th17 ratio was negatively correlated with the serum ACE level in the relapsed patients (r = -0.52, p<0.001), while the Treg/Th17 ratio showed a moderately positive correlation with a decrease in the FEV1% predicted value (r = 0.51, p<0.001). The Treg/Th17 ratio was moderately correlated with a decrease in the DLco% predicted value (r = 0.50, p<0.001, Fig 3).

### 6. The proliferation of Treg cells isolated from PBMCs recovered after sarcoidosis remission

**Fig 4:**
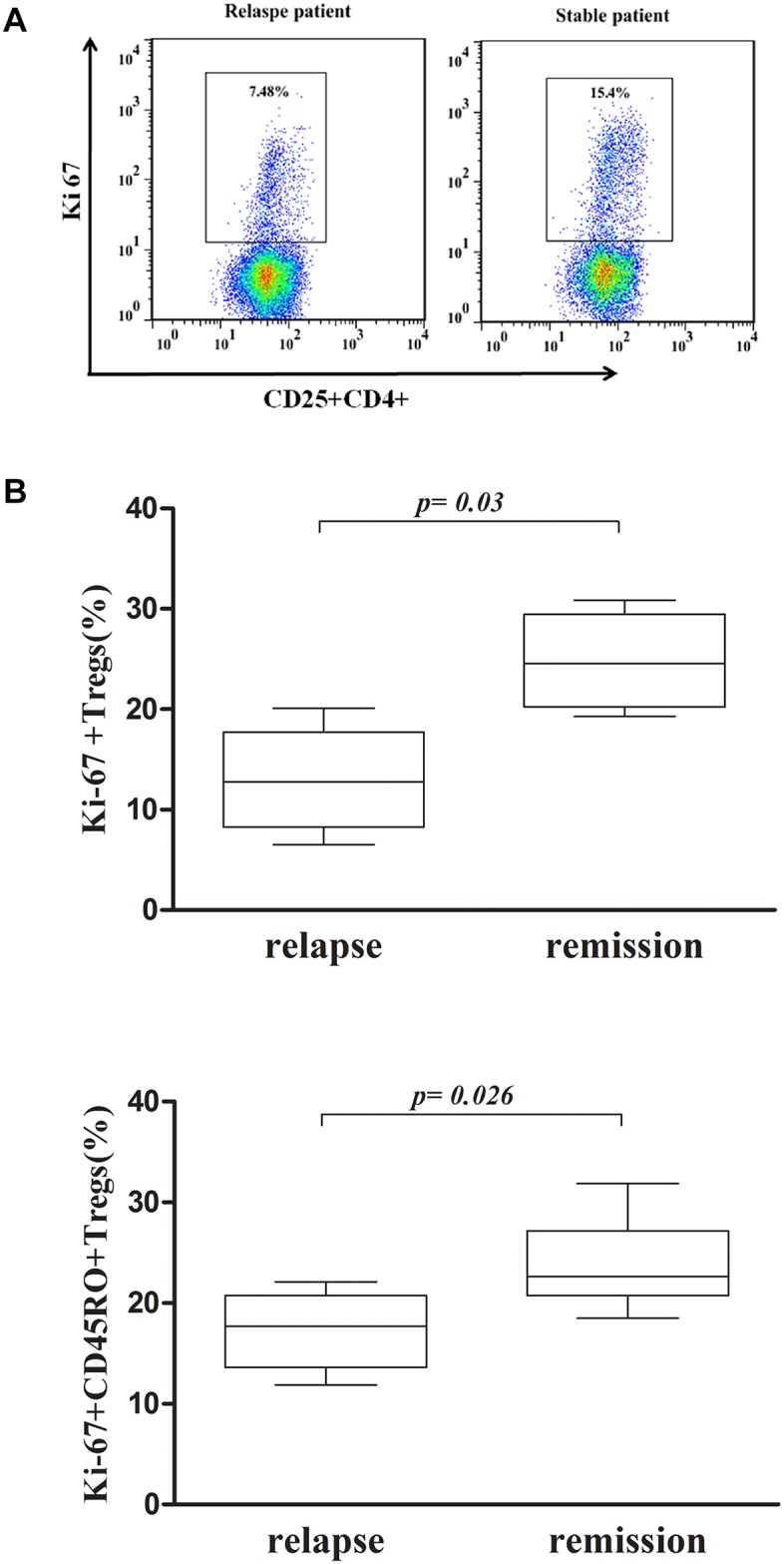
Proliferative activity of Tregs isolated by MACS via *in vitro* culturing. (A) Representative dot plots of Ki-67+ Treg cells from the relapsed and stable patients. The stable patients showed a higher percentage of Ki-67+ Tregs compared with the relapsed patients. (B) Proliferative activity of Tregs in six relapsed patients. Activity at the onset of relapse was compared with activity after retreatment. The percentage of Ki-67+ Tregs was increased after retreatment, and that of CD45RO+Ki-67+ Tregs was increased after remission.

Treg proliferative activity might be an indicator of the inhibitory functions of these cells. Therefore, we considered Ki-67 to be a marker of Treg proliferation and analyzed the percentage of Ki-67-expressing cells among the isolated Tregs by performing *in vitro* culturing. The percentage of Ki-67+ Treg cells was higher in the stable patients than in the relapsed patients (26.5±2.0% vs. 16.7±1.2%, p = 0.005, Fig 4A). Tregs were isolated from six relapsed patients by MACS at the onset of relapse and after 12 weeks of retreatment, and the purity of these isolated Tregs was above 90%. The percentage of Ki-67-positive Tregs out of the total number of Tregs was higher after remission (12.98±5.09% vs. 24.8±4.6%, p = 0.03), and the percentage of these cells out of the total number CD45RO+ Tregs accounted for most of this increase (17.32±3.84% vs. 23.8±4.5%, p = 0.026, Fig 4B).

### 7. Measurement of the Treg/Th17 ratio, serum ACE, and FEV1% and DLco% predicted values during dynamic follow-up in pulmonary sarcoidosis patients

**Fig 5:**
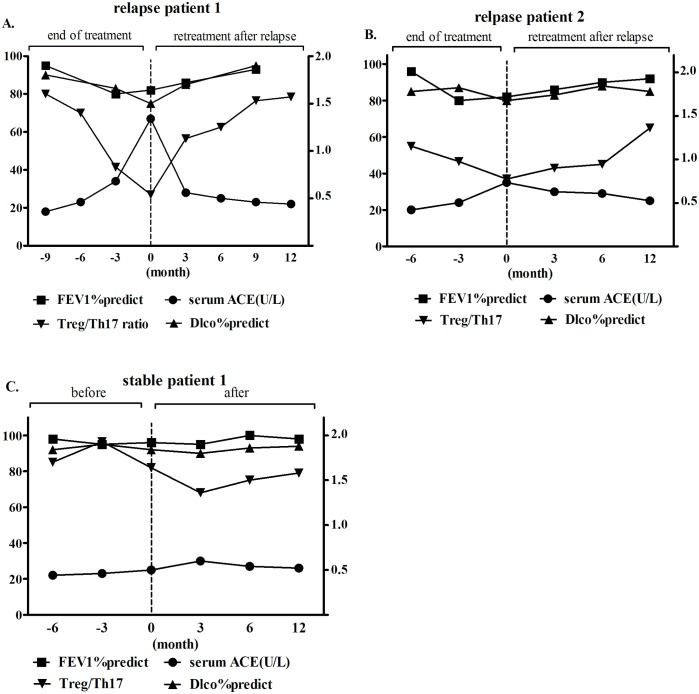
Dynamic follow-up of the Treg/Th17 ratio, serum ACE and FEV1% and DLco% predicted values in pulmonary sarcoidosis patients. (A) The Treg/Th17 ratio decreased, accompanied by an elevation in the serum ACE level and decreases in the FEV1% and DLco% predicted values over the nine months of follow-up after the end of treatment in the first relapse patient; then, the Treg/Th17 ratio dramatically increased, accompanied by a decrease in the serum ACE level and increases in FEV1% and DLco% predicted values over the first 3 months of retreatment. Over the 12 months of follow-up, these values stabilized. (B) The Treg/Th17 ratio decreased over 6 months of follow-up after the end of treatment, without distinct changes in the serum ACE or FEV1% and DLco% predicted values in another relapse patient; then, the Treg/Th17 ratio slightly increased over the first 3 months of retreatment, with symptom remission at 12 months of follow-up. (C) The Treg/Th17 ratio, serum ACE and FEV1% and DLco% predicted values remained stable before the end of prednisone treatment in a stable patient; all of these parameters remained stable without clinical symptoms over the subsequent 12 months.

To estimate the value of the Treg/Th17 ratio in long-term sarcoidosis treatment, we followed up three patients who exhibited good compliance for at least one year, and we collected blood samples regularly; these three patients included two relapse patients and one stable patient. The first relapse patient showed a decreased Treg/Th17 ratio, in accordance with the elevated sACE and decreased FEV1% and DLco% predicted values, over the nine months of follow-up after the end of prednisone treatment. Subsequently, the Treg/Th17 ratio dramatically increased, accompanied by a decrease in serum ACE and improvements in the FEV1% and DLco% predicted values during the first three months of retreatment; these values were stable at twelve months of follow-up (Fig 5A). However, the Treg/Th17 ratio decreased at six months of follow-up after the end of treatment without distinct changes in the serum ACE or FEV1% and DLco% predicted values in another relapse patient; then, the Treg/Th17 ratio slightly increased during the first three months of retreatment, accompanied by symptom remission and radiographic improvement over 12 months of follow-up (Fig 5B.). The Treg/Th17 ratio, serum ACE, and FEV1% and DLco% predicted values, symptoms, and radiography results did not change before the end of treatment in the stable patient, and all of these parameters were stable without symptom recurrence over the subsequent 12 months of follow-up (Fig 5C).

## Discussion

To date, few prospective studies have been performed to assess the immune statuses of relapsed pulmonary sarcoidosis patients after the end of treatment. Relapse has been found to affect patients’ the quality of lives and psychosocial burdens in clinical practice. In this study, we investigated a sarcoidosis patient cohort treated at our center after corticosteroid withdrawal. Nearly half of the sarcoidosis patients relapsed after corticosteroid withdrawal, and these patients demonstrated symptom recurrence or radiological progression, indicating that relapse was frequent after the end of corticosteroid treatment. Corticosteroids act by reversing the granulomatous inflammation process; thus, their effects are only immunosuppressive. However, it remains uncertain whether they can directly prevent the development of fibrotic lesions and maintain the disease in a stable state [27]. In most studies, the definitions of relapse have been vague, and recurrence of the disease could be considered as an acute exacerbation or reactivation of sarcoidosis in the thorax [28]. In other conditions, the disease activity might have never truly been under control; therefore, the manifestations became more apparent during anti-inflammatory drug withdrawal [29]. Interestingly, one of the risk factors associated with a high rate of sarcoidosis relapse has been reported to be the previous use of corticosteroids [21]. In the present study, we compared the clinical characteristics of relapsed patients with those of stable patients after medication withdrawal. The relapsed patients had a short duration of prednisone therapy, which was due to poor compliance or concerns about the side effects of long-term corticosteroid use. Most of the relapsed patients presented with repeated coughing or exacerbation of dyspnea, accompanied by recurrence with organ involvement, such as skin erythema or uveitis. A radiological investigation may aid in confirming the relapse of sarcoidosis based on lesion progression in the lung field or mediastinum. The inflammatory activity index was elevated, and PFT parameters were decreased significantly in some relapsed patients. Additionally, serum ACE was only elevated in some of the relapsed patients, which may be in accordance with the progression of the disorder.

As mentioned above, CD4+ T cell homeostasis plays important roles in granulomatous inflammatory activity and spontaneous resolution of sarcoidosis. Evidence of an elevated CD4+/CD8+ T cell ratio in BALF might be an important guiding factor in sarcoidosis diagnosis [30]. However, it might be impractical to predict relapse by replicating bronchoscopic examinations because of patients’ non-acceptance, and some cases of sarcoidosis relapse only present as enlarged mediastinal lymph nodes. Therefore, in this study, we intended to investigate the peripheral immunological markers that might be effective for managing sarcoidosis relapse and retreatment. Early studies showed that the Th1/Th2 ratio in BALF and PBMCs from patients demonstrated dynamic variations, which might play vital roles in the pathogenesis of active sarcoidosis [31]. However, the nature of the antigens that initiate Th1 responses in sarcoidosis and the mechanisms leading to the dissemination of granulomas and fibrosis remain unknown. Corticosteroid therapy has been reported to restore the normal balance of serum Th1/Th2 cytokines in sarcoid lungs in a cohort in which a limited number of patients were treated with steroids, which were added on the basis of previous antituberculosis therapy [32]. Unexpectedly, on the cellular level, there were no differences in CD4+ helper T cells, which produce IFN-γ and IL-4, between the relapsed and stable patients in our study. We postulate that this finding may be due to the fact that our enrollment criteria excluded patients who suffered from tuberculosis in the past, as it may be difficult to distinguish some pulmonary sarcoidosis patients from tuberculosis patients, and infection with *Mycobacterium tuberculosis* may influence the Th1/Th2 balance. We assumed that either (1) Th1 and Th2 cells may not be the key factors involved in maintaining sarcoidosis in a stable state or (2) the Th1/Th2 balance is not affected in patients who have previously undergone treatment with corticosteroids. The functions of the Th1 subset in relapsed patients who were at the end of corticosteroid treatment were different from those in patients during the early stage of granuloma formation. Additionally, this subset remained stable during retreatment and was not useful for assessing relapsed patients. In addition to other reasons, this finding might be due to the limited sample size or the short duration of the study. Some studies have shown that the IFN-γ+IL-17A+ subset, which is defined as the Th17.1 subset, is highly expressed in the BALF and lymph nodes of sarcoidosis patients compared with healthy controls in separate cohorts [33,34]; however, the IFN-γ+IL-17A+ subset in the peripheral blood did not differ between the patients who relapsed and those who were undergoing retreatment in this study.

The occurrence of uncontrolled inflammatory responses in sarcoidosis suggests that the immunoregulatory process might be impaired. In our study, we found that the frequency of Tregs was increased in parallel with an increase in Th17 cells in the relapsed patients compared with the stable patients. Treg cells comprise a family of regulatory cells with two subsets, namely naïve Tregs and induced Tregs, the latter of which are primed by self or exogenous antigens. TGF-β induces the Treg-specific transcription factor Foxp3, which is required for the induction and maintenance of induced Treg cells in the peripheral immune compartments [35]. In agreement with our previous study, which showed global amplification of Tregs in active sarcoidosis and large numbers of these cells in the peripheral blood and BALF [17], we found that their percentage was increased in the relapsed patients compared with the stable patients in this study. However, Foxp3 expression was lower in the patients who were stable than in those with active sarcoidosis who were not treated with corticosteroids. Other studies have reported some contradictory results with respect to the frequency of Tregs in pulmonary sarcoidosis [36], which may be due to differences in enrollment criteria or the immunological statuses of the studied patients. Th17 cell-mediated immune responses are important, not only in host defense but also in promoting chronic inflammation. A few studies have analyzed the roles of the Th17 effector cytokines IL-17A/F, IL-21, and IL-22 in the pathogenesis of sarcoidosis. The elevated level of IL-17A+CD4+ T cells in the peripheral blood of relapsed patients indicated the persistence of chronic inflammatory activity; a decrease in this activity was accompanied by remission of the disease. Otherwise, the reciprocal relationship between Th17 cells and Tregs is complicated, as these two types of cells might originate from the same naïve T cells in different immunological environments. The participation of TGF-β in the differentiation of Th17 cells also induces the differentiation of naïve T cells into Tregs in the peripheral immune system. At the functional level, TGF-β induces the generation of Tregs, which inhibits inflammation, maintains self-tolerance in the steady state, and prevents Th17 cell generation, leading to inhibition of the proinflammatory response. At the molecular level, the balance between Tregs and Th17 cells is maintained by the induction of Foxp3 and RORγt [37]. The dampening of Th17-driven immunopathology is believed to promote the generation of Foxp3+ Tregs, and this process is a novel target for the control of sarcoidosis and therapeutic surveillance. Maintenance of a balance between Tregs and Th17 cells may ensure that sarcoidosis remains in a stable state after corticosteroid withdrawal.

We next investigated the reciprocal relationship between Tregs and Th17 cells during retreatment. We found that the total number of Tregs was increased in accordance with a decrease in Th17 cells after retreatment in the relapsed patients; thus, we hypothesized that the balancing of Th17 cells and Tregs allows for the maintenance of homeostatic control mediated by immunoregulation in sarcoidosis. Tregs play a role in the remission of inflammation; thus, they may also have a role in the assessment of retreatment efficacy. Due to the parallel changes in Treg cells and Th17 cells, we considered the ratio of Treg/Th17 cells to be an index of advanced analysis, and we observed a significantly elevated ratio in the remission patients who underwent retreatment. In addition, this ratio was correlated with the serum ACE and PFT parameters. The Treg/Th17 ratio was demonstrated to indirectly reflect inflammation in pulmonary sarcoidosis in accordance with symptom severity. The relapsed patients in this study mainly showed clinical manifestations such as cough and dyspnea, with distinct decreases in the FEV1% and DLco% values compared with the stable patients. These decreases were caused by airway inflammation associated with airway physiological dysfunction and alveolar diffusion disorders. The Treg/Th17 ratio may be useful for assessing the inflammation mediated by immune factors in these patients and may be related to impairments in pulmonary function. Serum ACE is elevated in some patients with pulmonary sarcoidosis, and this parameter can be valuable for diagnosis; however, ACE dynamics may not completely reflect the treatment status. Additionally, a serum ACE level that is within the normal range may not be predictive of true relapse; however, the reasons for this are unknown. Therefore, the Treg/Th17 ratio, as a biomarker, might indirectly reflect the severity of relapse and might assist in clinical diagnosis and in predicting remission after retreatment.

Our results have shown that the percentage of Tregs in relapsed patients restored after remission. In a recent study, the survival of Tregs was impaired in active sarcoidosis patients compared to healthy controls, which may support the notion that Treg proliferation and apoptosis have indirect roles in sarcoidosis [38]. Previous reports have shown that CD25 bright Tregs mainly suppress the proliferation of inflammatory cells, leading to local immune anergy [39]. CD25 is composed of an α chain conjugated to IL-2, and a high level of CD25 expression indicates the activation of Tregs. However, Tregs with brighter CD25 were not the main contributors to relapse in our study. At the same time, the functions of Tregs in the remission and stable patients were associated with enhanced proliferation. Ki-67 is a marker of nuclear proliferative activity in the cell cycle, and we found that Ki-67+ Tregs were dominantly expressed in both the stable and remission patients after retreatment. CD45RA- Tregs have been reported to constitute the main subset during granuloma formation in a previous study [40], and other studies have shown that memory CD4+ T cells promote the remission of sarcoidosis. Memory Tregs might recall the antigen response corresponding to granulomatous inflammation [41]. Interestingly, CD45RO+Ki-67+ Tregs isolated from PBMCs were dominant in our study, indicating that this memory population was a key factor for the homeostasis of sarcoidosis. We postulate that CD45RO+ Tregs play a role in regulatory function after corticosteroid treatment.

In addition, our findings demonstrate the advantage of monitoring the Treg/Th17 ratio during long-term follow-up before and after corticosteroid treatment. Based on analysis of the Treg/Th17 ratio throughout the regular follow-up period in several highly compliant patients, we found that dynamic changes in this ratio could reliably predict the possibility of relapse after corticosteroid withdrawal or remission after retreatment, especially in relapsed patients without significant fluctuations in serum ACE or PFTs. We postulated that this ratio could be useful in relapsed patients prior to the onset of clinical manifestations.

Unfortunately, our study was limited because we assessed only peripheral blood immunophenotypic characteristics rather than the characteristics of BALF cells. Lymphocytes in BALF drained from the local lung lymph nodes can be used to more accurately interpret outcomes. In addition, the scale of our study was limited, and extension of the inspection phase from the end of corticosteroid treatment will be needed in further studies. Corticosteroids should also be compared with other novel medication agents. The association between the Treg/Th17 ratio and corticosteroids in refractory patients may also be examined in further studies.

In conclusion, our results have demonstrated that an imbalance between Treg cells and Th17 cells might play an important role in sarcoidosis relapse after corticosteroid withdrawal and that the Treg/Th17 ratio is correlated with relapse and remission in sarcoidosis patients. CD45RO+ Treg proliferative activity might be impaired during relapse after corticosteroid withdrawal. Our findings suggest that the Treg/Th17 ratio is a suitable biomarker for predicting sarcoidosis relapse combined with other clinical indications. Thus, it is worthwhile to monitor this ratio at the end of corticosteroid treatment and to conduct regular follow-ups.

## Supporting Information

S1 Fig Pulmonary sarcoidosis patients enrollment and follow-up. (TIF)
