# Supplementary figures and images for: The Circulating Treg/Th17 Cell Ratio Is Correlated with Relapse and Treatment Response in Pulmonary Sarcoidosis Patients after Corticosteroid Withdrawal
Source: PLoS One. 2016 Feb 4;11(2):e0148207. doi: 10.1371/journal.pone.0148207 (PMC4742270; doi:10.1371/journal.pone.0148207)

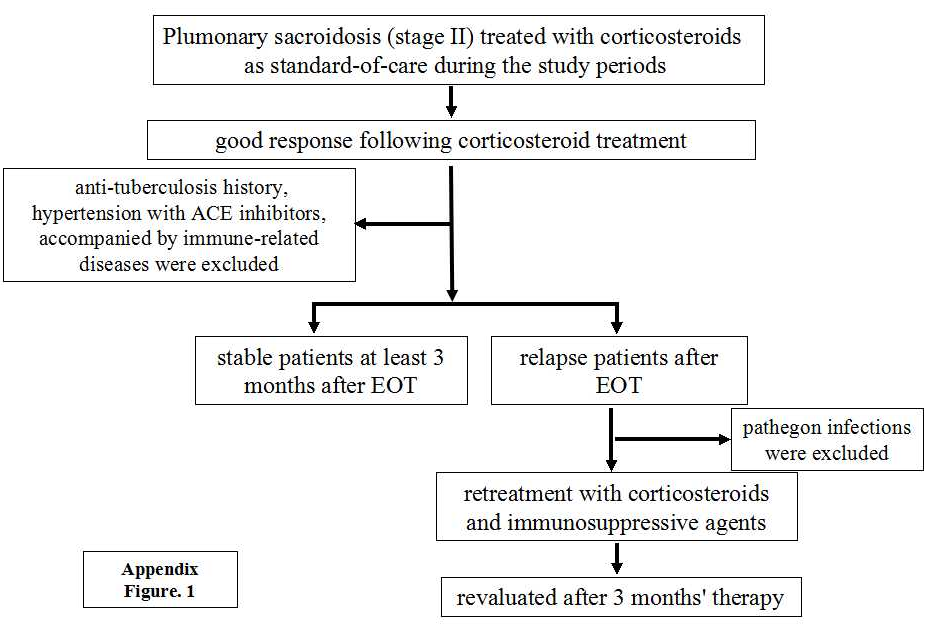

Supplement: S1 Fig — (TIF) [file pone.0148207.s001.tif]
